# The Biomechanical Characterization of the Turning Phase during a 180° Change of Direction

**DOI:** 10.3390/ijerph18115519

**Published:** 2021-05-21

**Authors:** Enrico Santoro, Antonio Tessitore, Chiang Liu, Chi-Hsien Chen, Chutimon Khemtong, Mauro Mandorino, Yi-Hua Lee, Giancarlo Condello

**Affiliations:** 1 Department of Movement, Human and Health Sciences, University of Rome Foro Italico, Piazza Lauro De Bosis 6, 00135 Roma, Italy; enry9521@gmail.com (E.S.); antonio.tessitore@uniroma4.it (A.T.); mauromandorino@gmail.com (M.M.); 2 Institute of Sports Equipment Technology, University of Taipei, 101 Zhongcheng Rd. Section 2, Shilin District, Taipei 111, Taiwan; chiangliu1974@yahoo.com.tw (C.L.); 3 Graduate Institute of Sports Training, Institute of Sports Sciences, University of Taipei, 101 Zhongcheng Rd. Section 2, Shilin District, Taipei 111, Taiwan; tmedmet.md_sci3@hotmail.com; 4 Department of Ball Sports, University of Taipei, 101 Zhongcheng Rd. Section 2, Shilin District, Taipei 111, Taiwan; lykllyy@utaipei.edu.tw

**Keywords:** modified 505 test, kinetic variables, completion time, foot contact, predictors

## Abstract

The aim of this study was to characterize the turning phase during a modified 505 test. Forty collegiate basketball students, divided into faster and slower performers and high-playing-level and low-playing-level groups, were evaluated for the force-time characteristics (braking and/or propulsive phase) of the penultimate foot contact (PFC), final foot contact (FFC), and first accelerating foot contact (AFC), and for completion time and approach velocity. Based on the composition of the AFC, trials were classified as braking/propulsive or only propulsive. Regression analysis for the prediction of completion time was performed. The AFC contributed to reacceleration through shorter contact times and step length, and lower braking force production (*p* < 0.05). Faster performers and the high-playing-level group demonstrated (*p* < 0.05): lower completion times, higher approach velocities, longer steps length in the PFC and FFC, greater braking forces and impulses in the PFC; greater braking and propulsive forces, braking impulses, lower contact times in the FFC; greater braking and propulsive horizontal forces, horizontal impulses, lower contact times and vertical impulses in the AFC. Kinetic variables from only the FFC and AFC and approach velocity predicted 75% (braking/propulsive trials) and 76.2% (only-propulsive trials) of completion times. The characterization of the turning phase demonstrated the specific contribution of each foot contact and the possible implications for training prescription.

## 1. Introduction

Change-of-direction (COD) ability is a preplanned, multidirectional action and an important physical quality for many team sports [1,2,3]. It can be defined as the ability to decelerate (i.e., eccentric action) in the shortest time and quickly reaccelerate (i.e., concentric action) in a new direction while running or sprinting [1,2,3,4]. This coupling of an eccentric and concentric action also refers to the ability to properly use the stretch-shortening cycle (SSC) [5].

A COD can be performed with or without a stimulus to which to respond. Specifically, when a directional change occurs in response to a stimulus, it is referred to as agility, while it is only referred to as COD speed if the response to a stimulus is not required [1,2,3]. Focusing solely on the preplanned action during training and competitions, CODs are executed at different directions, speeds, and with different cutting angles. In particular, a 180° COD action frequently occurs during competitions (i.e., basketball, soccer, netball, cricket) [6,7,8]. For this reason, it is widely included in fitness testing batteries for either COD speed tests (i.e., 505 test) or endurance field-based cardiorespiratory tests (i.e., Yo-Yo test, 30–15 Intermittent Fitness Test). In this context, the test’s completion time is mainly used as the performance outcome. However, an exhaustive evaluation of the COD ability requires technical and biomechanical assessments [9,10,11,12], hence focusing on the “how” (i.e., quality) and not only on the “what” (i.e., time) of the COD performance to better address training prescription [13]. Therefore, the investigation of the “how” has been consistently addressed through the assessment of kinetic and kinematic variables during the critical foot contacts of COD performance [14].

Focusing solely on the 180° COD speed tests, the kinetic and kinematic variables of the penultimate foot contact (PFC) and final foot contact (FFC) are commonly evaluated during traditional and modified 505 tests to investigate the differences between faster and slower performers [15,16,17]. Recently, new research investigated for the first time the antepenultimate foot contact, demonstrating its role in facilitating the deceleration phase during a traditional 505 test [18]. However, it would be interesting to also consider the analysis of the first step after turning, henceforth called the first accelerating foot contact (AFC). However, to the best of our knowledge, previous studies did not include the analysis of this foot contact during traditional or modified 505 tests.

The available evidence demonstrates the differences between faster and slower performers (based on the completion time) during the 505 test. Faster performers showed greater horizontal braking forces for the PFC, greater horizontal propulsive forces, vertical impact forces, and shorter contact times for the FFC compared with slower performers during the modified 505 test [15]. Furthermore, faster performers demonstrated greater vertical braking and propulsive forces compared with slower performers in the COD-only step analyzed during the traditional 505 test [16]. Moreover, faster performers showed greater peak and average horizontal propulsive forces for the FFC than slower performers during the traditional and modified 505 test [17]. Finally, associations have recently been demonstrated between greater antepenultimate foot contact peak vertical, horizontal, and resultant braking forces, mean vertical, horizontal, and resultant GRFs, and horizontal total impulses in comparison with faster performers during the traditional 505 test [18].

Furthermore, the possible influence of limb asymmetries and directional dominance might exist in the traditional or modified 505 test in female and male team sports players [19,20]; however, it has not been largely confirmed in a further investigation of only female soccer players [21]. Nonetheless, contradictory results also emerged considering limb dominance as a factor associated with injury risk during COD actions [22].

Playing level might be considered a critical issue in sports performance. However, it has been demonstrated that COD speed tests are not able to discriminate between higher-skilled groups [3]. In fact, previous investigations did not report significant differences between higher- and lower-performance groups in Australian football [23,24,25] and rugby league [26,27] players when the completion time was measured. However, there is a paucity of evidence regarding the effect of the playing level on spatial–temporal and kinetic variables during a 505 test.

Similarly, considering that several determinants (spatial–temporal and kinetic variables) may influence the performance outcome (i.e., completion time) of a 505 test, limited evidence is available for the predictors of completion time.

Taken together, the current knowledge on the 505 test can still be expanded through the inclusion of other foot contacts with those already investigated in order to define the entire turning phase, as well as in relation to several factors, such as leg dominance, COD performance, and playing level. Therefore, the objective of this study was to investigate the modified 505 test, providing an analysis of the three foot contacts (PFC, FFC, AFC) that characterize the turning phase. In particular, the purpose of this study was to examine the effects of leg preference, COD performance, and playing level on spatial–temporal and kinetic variables for each foot contact and to evaluate the prediction of the completion time from the spatial–temporal and kinetic variables. We hypothesized the existence of differences between legs and superior performance for faster performers and the high-playing-level group during the modified 505 test.

## 2. Materials and Methods

### 2.1. Study Design

A cross-sectional study design was applied to investigate the effect of leg preference, COD performance (i.e., completion time), and playing level on spatial–temporal and kinetic variables during the modified 505 test. Furthermore, the predictors of COD performance were examined.

This study was approved by the University of Taipei Institutional Review Board (Taipei, Taiwan, reference number: IRB-2018-093). All participants gave their informed written consent, and all the experimental procedures were conducted in accordance with the Declaration of Helsinki [28].

### 2.2. Participants

A minimum sample size of 34 participants was determined from an a priori power analysis performed by G*Power (version 3.1.9.2 University of Dusseldorf, Dusseldorf, Germany), considering an ANOVA test with a power of 0.95, an effect size of 0.65, and α = 0.05. Accordingly, 40 male and female collegiate students (32 males: age = 20.9 ± 2.0 years, height = 179 ± 7.3 cm, body mass = 76.1 ± 9.6 kg; 8 females: age = 21.5 ± 1.8 years, height = 164 ± 8.4 cm, body mass = 58 ± 8.6 kg) were recruited to participate in this study and were eligible in accordance with the following inclusion criteria: (a) age 18–25 years; (b) absence of known cardiovascular, pulmonary, metabolic, bone, or joint diseases; (c) no smoking; (d) no muscle and joint injuries during the last six months. Participants were asked to identify their preferred leg used to kick a ball, which was then identified as the kicking leg (KL). Consequently, the opposite leg was ascertained to be the leg used to jump off when performing a right-handed running basketball layup and was identified as the stance leg (SL) [29]. Participants were divided into faster (top 33%, *n* = 13) and slower (bottom 33%, *n* = 13) performers based on their completion time [17] to test the hypothesis of the effect of COD performance. In addition, participants were divided into a high-playing-level group (*n* = 17), engaged in basketball training and competitions at collegiate and national levels (>3 training sessions per week; >5 years of basketball experience), and a low-playing-level group (*n* = 23), engaged in basketball as a recreational activity (<3 sessions per week), to test the hypothesis of the effect of playing level.

### 2.3. Procedures

Participants reported to the laboratory on two occasions separated by a 72 h resting period at the same time of the day (10:00 ± 30 min), with temperature and humidity kept consistent at 24 ± 1 °C and 55 ± 5%, respectively. They were required to abstain from exercise during the 72 h prior to each experimental session and to abstain from alcohol and caffeine consumption during the 12 h prior.

After ascertaining the inclusion criteria, participants were familiarized with all the experimental procedures during the first experimental session. Moreover, height (cm) and body mass (kg) were measured to the nearest decimal using a Jenix DS-102 stadiometer (Dong Sahn Jenix Co., Ltd., Seoul, South Korea). During the second experimental session, participants performed the modified 505 test (Mod505) [26], in which they were required to sprint forward for 5 m, make a 180° COD while on the force plates, and sprint back for another 5 m. Participants were instructed: (a) to start 0.5 m from the start line with their preferred foot forward in a two-point stance; (b) to have a straight trajectory toward the force plates; (c) to make the COD with the external leg on a visual target (X) highlighted on the middle of the second force plate; (d) to exert maximal effort during the entire course of the test. Participants performed several trials (5 to 7) for each leg (alternating one trial for each leg) with a 2 min resting period in between. A trial was included in the analysis if the participants had a straight trajectory toward the force plates without prior stuttering or prematurely turning prior to final contact and made full contact with the force plates during the three foot-contacts of the turning phase [30]. Inspection of full contacts was performed at the end of each trial using two video cameras synchronized with the Optojump photoelectric system and the force plate software showing the pushing area of the foot. The fastest four trials were considered suitable for analysis and the average value for each investigated variable was used for the statistical analysis. 

Participants used the same model of basketball shoes (Adidas Pro Bounce 2019, Herzogenaurach, Germany) to reduce the variability given by the use of different types of sports shoes. Before the experimental session, they completed a standardized warmup involving 3 min of jogging on a treadmill followed by dynamic stretching, squats, frontal and lateral lunges, short accelerations, directional changes, and submaximal trials of the test.

The experimental protocol was executed in a laboratory setting (Figure S1) with the simultaneous use of two adjacent embedded force plates with a sampling rate of 2400 Hz (60 cm × 90 cm; BMS 600900 OPTIMA™ Biomechanics Measurement Series, AMTI, Watertown, MA, USA) and an Optojump photoelectric system (OptojumpNext, Microgate, Bolzano, Italy) placed beside the force plates, covering the entire 5 m course. The transmitter and receiver bars were placed 2 m apart. Force plates were covered with anti-slip tape to prevent slippage. Moreover, a set of a timing lights system (SMARTSPEED; Fusion Sports, Queensland, Australia) was placed in correspondence with the start/stop line at the hip height of participants to avoid other body parts (unless the lower torso) activating the infrared light.

### 2.4. Data Processing

Due to the use of two adjacent force plates, the definition of the turning phase from one direction to the new direction was achieved, consisting of three consecutive foot contacts: (1) the PFC, defined as the second last foot contact with the first force plate before moving towards a new intended direction [17]; (2) the FFC, defined as the turning foot while in contact with the second force plate to initiate the movement towards a new intended direction [17]; and (3) the AFC, defined as the first accelerating foot contact with the first force plate moving in the new intended direction. The PFC and FFC have been previously investigated [14,17,19], whilst the AFC was first evaluated in this study. A preliminary analysis was completed to verify the composition of each foot contact, using the resultant GRF. Accordingly, the PFC consisted only of a braking phase, whilst the FFC consisted of both the braking and propulsive phases, as already demonstrated in previous research [14,17,19]. In contrast, two executions have been identified for the AFC, with some trials characterized by both braking and propulsive phases and some trials encompassing only a propulsive phase. Therefore, considering the execution of the AFC, each trial for every participant was categorized as a braking/propulsive trial or only-propulsive trial and was included in the analysis in an attempt to explain possible differences for the investigated variables.

**Figure 1:**
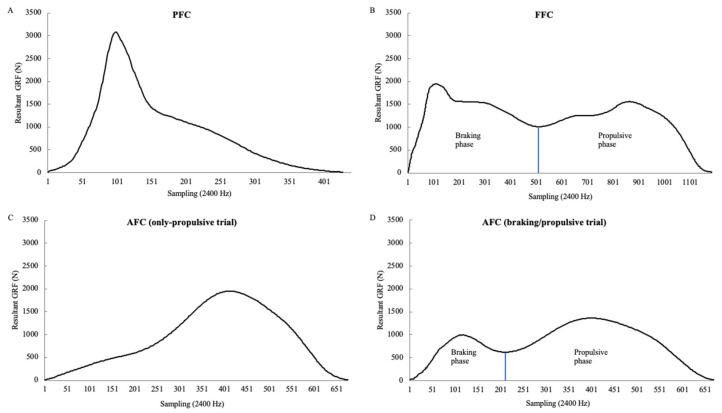
Example of vertical ground reaction force (GRF) profiles for (**A**) penultimate foot contact (PFC), (**B**) final foot contact (FFC), and first accelerating foot contact (AFC) in case of (**C**) only-propulsive trials and (**D**) braking/propulsive trials, used for the identification of the braking and propulsive phases.

Data from force plates were collected using Cortex software (version 3.6.0; Motion Analysis Corp., Santa Rosa, CA, USA), digitally filtered at 25 Hz using a Butterworth low-pass filter, and imported and analyzed with Microsoft Excel (Microsoft Corp, Redmond, WA). The three components of the GRF were vertical (Fz), anterior-posterior (horizontal; Fx), and mediolateral (Fy). Foot contact was defined from the initial contact (touchdown), when the vertical GRF was above a threshold of 10 N, to the final contact (takeoff), when the vertical GRF was below a threshold of 10 N [10,31]. The identification of the braking and propulsive phases was based on the bimodal resultant GRF profile. The braking phase spans from the initial contact to the minimal value between the two peaks, while the propulsive phase spans from the minimal value between the two peaks to the takeoff. Therefore, for AFC classification, the braking/propulsive trials were characterized by a bimodal GRF profile with two peaks, whilst the only-propulsive trials were characterized by a unimodal GRF profile with a single peak (Figure 1).

The variables obtained from the force plates for each foot contact (for either braking, propulsive, or both phases) included: braking, propulsive, and total contact time (CT); peak relative braking and propulsive GRF for both vertical and horizontal components (VGRF and HGRF); relative braking, propulsive, and total impulse for both vertical and horizontal components (VImp and HImp). All GRF and impulse variables were normalized by body mass. Moreover, peak braking and propulsive resultant GRFs were calculated using the Pythagorean theorem [17]:

$$\mathrm{resultant}\;\mathrm{force}=\sqrt{\left({\mathrm{vertical}\;\mathrm{force}}^{2}\right)+({\mathrm{horizontal}\;\mathrm{force}}^{2}})$$

The variables derived from the Optojump system (directly provided by the dedicated software, version 1.12.15, OptojumpNext, Microgate, Bolzano, Italy) were step length and approach velocity. Step length was calculated as the tip-to-tip distance from one foot to the next (i.e., right–left, left–right). According to the manufacturer’s instructions, velocity was calculated as V = L/(Tc + Tf), where L is step distance, Tc is contact time, and Tf is flight time; all parameters were previously investigated for their reliability in gait analysis [32]. Therefore, approach velocity referred to the velocity at the last foot contact before the turning phase. A preliminary assessment of the data among trials revealed that approach velocity at the last foot contact before the turning phase was the last one with increasing velocity, then the PFC (i.e., the first foot contact of turning phase) consistently showed a decrease in velocity.

Finally, the completion time was measured to the nearest 0.01 s and used as the outcome of COD performance.

The measurement of the investigated variables demonstrated “moderate” to “excellent” internal consistency reliability, ascertained by intraclass correlation coefficients (two-way mixed effects, average measures, absolute agreement). Intraclass correlation coefficients with the 95% confidence intervals are reported in Table S1 in the Supplementary Materials. Furthermore, a recent investigation demonstrated the concurrent validity and internal consistency reliability for the use of the force plate and Optojump system in evaluating the sprint test with a 180° COD [31].

### 2.5. Statistical Analysis

Data were analyzed using the Statistical Package for the Social Sciences, version 25.0 (SPSS Inc., Chicago, IL, USA). The level of statistical significance was set at *p* < 0.05 for all computations. The normality assumption for each variable was verified using the Shapiro–Wilk test, which confirmed the normal distribution of data.

Since prior analysis showed no gender differences for the investigated variables, data from male and female participants were pooled. Moreover, since the analysis for the stance and kicking legs did not reveal differences, the data were pooled to further increase the sample size. Statistics are provided in Table S2 of the Supplementary Materials.

Differences between braking/propulsive and only-propulsive trials were investigated with paired *t*-test. Cohen’s *d* effect sizes (ESs) were calculated and interpreted as trivial (<0.19), small (0.20–0.59), moderate (0.60–1.19), large (1.20–1.99), very large (2.0–4.0), and extremely large effects (>4.0) [33].

Independent sample *t*-tests were applied to ascertain differences between faster and slower performers and between the high-playing-level and low-playing-level groups for both braking/propulsive and only-propulsive trials. Hedges’ *g* effect sizes (ESs) were calculated and interpreted as trivial (<0.19), small (0.20–0.59), moderate (0.60–1.19), large (1.20–1.99), very large (2.0–4.0), and extremely large effects (>4.0) [33].

A stepwise multiple regression analysis was separately executed for the braking/propulsive and only-propulsive trials to create a model able to explain the prediction of the completion time from the spatial–temporal and kinetic variables. For braking/propulsive trials, the predictors included in the model were: step length, CT, braking VGRF, HGRF, VImp, and HImp for the PFC; step length, total, braking, and propulsive CT, braking and propulsive VGRF and HGRF, total, braking, and propulsive VImp and HImp for the FFC; step length, total, braking, and propulsive CT, braking and propulsive VGRF and HGRF, total, braking, and propulsive VImp and HImp for the AFC; approach velocity. 

For only-propulsive trials, the predictors included in the model were: step length, CT, braking VGRF, HGRF, VImp, and HImp for the PFC; step length, total, braking, and propulsive CT, braking and propulsive VGRF and HGRF, total, braking, and propulsive VImp and HImp for the FFC; step length, CT, propulsive VGRF and HGRF, VImp, and HImp for the AFC; approach velocity. Multicollinearity was ascertained using tolerance and the variation inflation factor (VIF) to verify the degree of correlations among the included predictors. Values lower than 0.20 for tolerance and higher than 10 for VIF denoted the presence of multicollinearity.

## 3. Results

### 3.1. Characterization of the Mod505 Performance and Turning Phase

A descriptive analysis of the entire course of the Mod505 revealed a total number of between 8 and 10 steps to complete the test. The shortest step length was for the AFC (80.6 ± 9.9 cm), whilst the last step was the longest (154.4 ± 23.4 cm). The approach velocity at the last foot contact before the turning phase was 5.40 ± 0.47 m/s. The turning phase lasted an average of 1.22 ± 0.17 s considering all the trials, representing 44.2% of the average completion time (2.77 ± 0.14 s). 

### 3.2. Analysis of Different Executions

Based on the execution of the AFC, the trials of every participant have been classified as braking/propulsive (65.8%) or only propulsive (34.2%). No differences emerged for completion time (braking/propulsive trials: 2.71 ± 0.13 s; only-propulsive trials: 2.73 ± 0.12 s), approach velocity (braking/propulsive trials: 5.46 ± 0.51 m/s; only-propulsive trials: 5.52 ± 0.46 m/s), and turning phase (braking/propulsive trials: 1.22 ± 0.17 s; only-propulsive trials: 1.23 ± 0.21 s). Significant differences (*p* < 0.05) between trials emerged for braking VGRF and resultant GRF in the PFC, braking and total VImp and braking HImp in the FFC, and propulsive VGRF and HGRF, total VImp, propulsive resultant GRF, and step length in the AFC (Table 1).

### 3.3. Analysis of COD Performance

Faster performers (completion time = 2.61 ± 0.07 s) revealed higher values for approach velocity (faster: 5.72 ± 0.42 m/s; slower: 5.13 ± 0.36 m/s; *p* < 0.001, ES = 1.51) compared with slower performers (completion time = 2.92 ± 0.06 s). For step length, differences between faster and slower performers emerged in the PFC (faster: 126 ± 32.2 cm; slower: 96.8 ± 31.4 cm; *p* = 0.005, ES = 0.92) and the FFC (faster: 95 ± 9.3 cm; slower: 85.2 ± 14.6 cm; *p* = 0.011, ES = 0.82) for the braking/propulsive trials, whilst only in the PFC (faster: 123.3 ± 31.2 cm; slower: 97.9 ± 37.6 cm; *p* = 0.042, ES = 0.75) for the only-propulsive trials, compared with slower performers A similar time for turning phase emerged for both braking/propulsive (faster: 1.19 ± 0.11 s; slower: 1.22 ± 0.13 s; *p* = 0.343; ES = −0.29) and only-propulsive (faster: 1.21 ± 0.14 s; slower: 1.18 ± 0.13 s; *p* = 0.556; ES = 0.22) trials. Significant differences (*p* < 0.05) between faster and slower performers emerged for kinetic variables and are presented in Table 2. For braking/propulsive trials, faster performers exhibited: (a) greater braking VGRF, HGRF, HImp, and resultant GRF in the PFC; (b) greater braking and propulsive HGRF, braking HImp, propulsive resultant GRF, lower propulsive and total CT, and propulsive and total VImp in the FFC; (c) greater propulsive HGRF, propulsive and total HImp, propulsive resultant GRF, lower propulsive CT, and propulsive and total VImp in the AFC, compared with slower performers. For only-propulsive trials, faster performers exhibited: (a) greater braking HGRF in the PFC; (b) greater braking and propulsive HGRF, braking and total HImp, and lower propulsive VImp in the FFC; (c) greater propulsive HGRF and HImp in the AFC, compared with slower performers.

### 3.4. Analysis of Playing Level

A significant difference emerged for height (high playing level: 181.4 ± 6 cm; low playing level: 172.1 ± 10 cm; *p* = 0.001; ES = 1.13), but not for body mass (high playing level: 76.4 ± 9 kg; low playing level: 69.5 ± 12 kg; *p* = 0.59; ES = 0.65).

The high-playing-level group demonstrated a shorter completion time (high playing level: 2.69 ± 0.14 s; low playing level: 2.82 ± 0.11 s; *p* < 0.001, ES = −1.05), a higher approach velocity (high playing level: 5.68 ± 0.44 m/s; low playing level: 5.19 ± 0.38 m/s; *p* < 0.001, ES = −1.21), and a longer step length for PFC (high playing level: 129.4 ± 29.5 cm; low playing level: 103.4 ± 31.6 cm; *p* < 0.001, ES = 0.85) and FFC (high playing level: 97.2 ± 7.4 cm; low playing level: 86.6 ± 10.8 cm; *p* < 0.001, ES = 1.12). A similar time for turning phase emerged for both braking/propulsive (high playing level: 1.20 ± 0.12 s; low playing level: 1.24 ± 0.17 s; *p* = 0.369, ES = −0.22) and only-propulsive (high playing level: 1.21 ± 0.16 s; low playing level: 1.23 ± 0.21 s; *p* = 0.672, ES = −0.12) trials. Significant differences (*p* < 0.05) between the high-playing-level and low-playing-level groups emerged for kinetic variables and are presented in Table 3. For braking/propulsive trials, the high-playing-level group exhibited: (a) greater braking VGRF, HGRF, VImp, HImp, and resultant GRF in the PFC; (b) greater braking and propulsive VGRF, propulsive HGRF, braking VImp, HImp, total HImp, and propulsive resultant GRF in the FFC; (c) greater propulsive VGRF and HGRF, braking HGRF, total HImp, propulsive and braking resultant GRF, and lower propulsive and total CT in the AFC, compared with the low-playing-level group. For only-propulsive trials, the high-playing-level group exhibited: (a) greater braking VGRF, HGRF, and resultant GRF in the PFC; (b) greater braking VGRF, VImp and HImp, total HImp, and braking resultant GRF in the FFC; (c) greater propulsive HGRF and HImp in the AFC, compared with the low-playing-level group.

### 3.5. Stepwise Multiple Regression Analysis

Table 4 shows the steps necessary to create the model for both braking/propulsive and only-propulsive trials. For braking/propulsive trials, model five has been identified to better predict the completion time, including the five predictors (i.e., FFC propulsive HGRF, AFC propulsive HGRF, FFC propulsive VGRF, AFC Total VImp, and AFC step length) that explain 75% of the common variance (Table 5). For the only-propulsive trials, model six has been identified to better predict the completion time, including the six predictors (i.e., approach velocity, FFC braking HGRF, FFC braking VGRF, AFC propulsive HGRF, FFC total CT, and AFC propulsive VGRF) that explain 76.2% of the common variance (Table 5). Data for correlations (partial and part) and collinearity analysis have been reported for the predictors included in both models for braking/propulsive and only-propulsive trials (Table 5). In particular, the lack of multicollinearity is demonstrated from the values higher than 0.20 for tolerance and lower than 10 for VIF for all the predictors included in the models.

## 4. Discussion

This study intended to examine the effects of leg preference, COD performance, and playing level and to explore the prediction of the completion time from spatial–temporal and kinetic variables. The main finding of this study is a superior performance of faster performers and the high-playing-level group (Table 2 and Table 3). Moreover, this study demonstrated for the first time the predictive variables of completion time (Table 5). The novelty of this study is the inclusion of the first accelerating foot contact in the analysis together with the penultimate and final foot contacts, revealing different executions. The lack of leg preference in making directional changes supports the controversy regarding the influence of interlimb asymmetries on sports performance. However, the inconsistency in evidence for interlimb asymmetries also depends on a lack of consensus regarding the definition and determination of leg preference and/or dominance [34,35].

A descriptive analysis shows that the AFC is characterized by lower values for contact time, braking GRF and impulse in both vertical and horizontal components, and resultant GRF compared with the PFC and FFC. Moreover, the AFC also revealed a shorter step length compared with the PFC and FFC. In particular, shorter contact times and step lengths might be expected to be necessary for the reacceleration in a new direction, given that AFC is the first foot contact after turning when the horizontal velocity of the center of mass is zero.

The finding of the different executions of the AFC was unexpected, with a higher proportion of the trials indicating braking before the propulsive phase, even though a full explanation has not been reached with this investigation. In fact, when braking/propulsive and only-propulsive trials were compared with spatial–temporal and kinetic variables (Table 1), marginal differences were found in the PFC and FFC, whereas in the AFC, higher values for propulsive vertical, horizontal, and resultant GRFs were found for the only-propulsive trials. However, the total vertical impulse was higher for the braking/propulsive trials, which can be explained by the further contribution of the impulse during the braking phase. Moreover, a difference in step length emerged between trials, with the braking/propulsive trials showing a longer AFC step length compared with the only-propulsive ones. Therefore, the presence of the braking phase could be attributed to the longer step length of the AFC. In fact, this longer step length may require a supporting base at the initial contact resulting in the application of the braking force. This action will also require the application of the SSC with the braking phase (i.e., eccentric action) acting to store elastic strain energy, which is subsequently recovered during the propulsive phase (i.e., concentric action) [36]. It is well-documented that the enhancement of sports performance can be achieved through the amplification of the force and power produced during the shortening cycle as a consequence of the previous elastic strain energy stored during the lengthening cycle [5,36]. In contrast, in the only-propulsive trials, the shorter step length allowed for the immediate exertion of the propulsive phase without the need to accumulate elastic energy in a braking phase. This may also reflect the existence of a different foot strike pattern between the two executions. It can be speculated that in the braking/propulsive trials, the foot contact comprised the rearfoot strike pattern, whilst a forefoot strike pattern was used in the only-propulsive trials [37]. Considering this first attempt to characterize the AFC and the observation of different executions, further research to confirm the first findings obtained by this study is highly recommended.

In line with previous research on COD performance [15,16,17,18], the current study demonstrated differences between faster and slower performers. The higher approach velocities for faster performers are in accordance with previous research on 505 tests [17] and athletes with higher eccentric strength [30]. Recently, approach velocity has been recognized as an important factor influencing COD performance, together with the angle of the COD (e.g., from 45° to 180°) [13]. Therefore, this study can contribute new evidence to the existing knowledge about approach velocity, using a photoelectric system for its determination. Further evidence is also provided for the step length, showing that a faster performance required longer steps length in the PFC and FFC, whilst no differences emerged for the AFC.

Regarding kinetic variables, several profiles and characteristics can be highlighted. For the PFC, the results demonstrate greater values for braking vertical and horizontal GRFs, horizontal impulse, and resultant GRF for faster performers, even though the contact times were similar. Therefore, for the same ground contact time, a higher force was exerted. Considering the higher approach velocities, these results might indicate that higher force production is necessary for the deceleration of the body [17]. Moreover, a higher force production has been associated with superior movement mechanisms and strength capacity, hence increasing the exit velocity during COD movements [16,38]. In particular, eccentric strength has been determined as the sole predictor of a 505 test in a sample of female basketball players [38]. Therefore, the single braking phase characterizing the PFC may explain the need for higher eccentric action to decelerate the body. For the FFC, higher values of horizontal GRF in both braking and propulsive phases, coupled with shorter total contact times, vertical propulsive, and total impulses, may confirm an efficient application of the SSC [17]. Considering that the FFC consisted of both braking and propulsive phases, faster performers might display superior ability in maximizing the application of the SSC, compared with their slower counterparts [17], based on the shorter ground contact times and impulses in the vertical component. Similarly, considering the braking/propulsive trials of the AFC, faster performers demonstrated lower values for contact time and vertical propulsive and total impulses, which can be required to accelerate the body. Therefore, the comparisons of kinetic variables between faster and slower performers may explain the action of foot contacts, with the PFC and FFC remaining critical foot contacts for reducing the momentum of the center of mass [15,19], whilst the AFC is important for the acceleration of the body in the new direction. 

Higher- and lower-skilled athletes have been commonly investigated for completion time, revealing COD speed tests are not able to discriminate playing level [23,24,25,26,27]. Conversely, in this study, the high-playing-level group had a lower completion time and a higher approach velocity. These results may have also determined the longer step lengths and the higher values for force-related variables in both the PFC and FFC compared with the low-playing-level group, even though similarities existed for the contact times. Greater force production during the braking phase in the PFC is required to start reducing the momentum of the center of mass due to the higher approach velocity [17]. However, contradictory results emerged in the FFC, with higher values for GRFs in both braking and propulsive phases, but also for impulses, which prevent the confirmation of a superior application of the SSC. Regarding the AFC, the high-playing-level group exhibited shorter contact times, as a result of the shorter propulsive phase, and higher values for GRFs during the propulsive phase, but only a difference for the total horizontal impulse. A superior reacceleration capacity in the new direction for the AFC is still speculated. However, the effect of playing level on spatial–temporal and kinetic variables may require further investigation to confirm the presented findings. 

The regression analysis was proposed to explore the prediction of the completion time from spatial–temporal and kinetic variables. Different models emerged for braking/propulsive and only-propulsive trials, comprising variables of the FFC and AFC, with ≥75% of the variance of completion time being explained. The propulsive horizontal GRF of the AFC was the only variable in common between the two trials. This result, combined with the higher propulsive horizontal GRF values found for faster performers and the high-playing-level group in both braking/propulsive and only-propulsive trials, might highlight the role of this variable as an important determinant of COD performance. The total vertical impulse and step length of the AFC were also included in the model for the braking/propulsive trials, together with propulsive horizontal and vertical GRF of the FFC. Therefore, the predictive analysis showed the importance of including the AFC in the assessment of a 180° COD test for a complete characterization of the turning phase. Conversely, none of the variables of the PFC were included in both models, even though the PFC has always been considered a critical determinant of the COD performance [14]. Moreover, recent research suggests that the antepenultimate foot contact might play a superior role in deceleration compared with the PFC [18]. Unfortunately, the antepenultimate foot contact was not included in the current investigation. However, we suggest that the turning phase should be investigated for all foot contacts composition. In summary, for braking/propulsive trials, an AFC characterized by a higher propulsive horizontal GRF and step length and a lower total vertical impulse, and an FFC characterized by a higher propulsive horizontal GRF and a lower propulsive vertical GRF, can predict shorter completion time. Conversely, for only-propulsive trials, an AFC characterized by a higher propulsive horizontal GRF and lower vertical GRF, and an FFC characterized by a higher braking horizontal GRF, a lower braking vertical GRF and total contact time, and a higher approach velocity, can predict shorter completion time. It might be speculated that approach velocity may play a role in discriminating between the two different executions and could determine the other kinetic variables since it entered the predictive model only for only-propulsive trials. Therefore, due to the differences in models and predictive variables, further research is necessary to fully explain the different executions identified in this study.

The present study has some limitations that need to be addressed and can serve as guidance for future research. The present findings cannot be extended to COD tests with a different degree of angle, since the COD performance is angle- and velocity-dependent [12]. Therefore, the turning phase can be investigated with different COD tests. Approach velocity was determined with a photoelectric system which, though reliable, is not as accurate as other methods. Future research can consider the measurement of approach velocity with trunk and lower limbs’ center of mass computation as proposed in previous investigations [17,30]. Moreover, 3D motion analysis has not been applied in the current study, limiting the investigation of kinematic determinants of the turning phase. It is strongly advised to replicate the current study design while adding 3D motion analysis. Although differences did not emerge between female and male participants, the different sample sizes between the two groups did not allow us to make definitive conclusions. It is advised to explore the gender differences using the same sample sizes. Similarly, all participants were basketball players, hence these findings cannot be generalized to other team sports players. Another limitation of this study, due to the laboratory setting, is the impossibility of really detecting different strategies used by participants and the potential interaction between dominance/preference and strategy. This is considered a critical issue when investigating preplanned action in a laboratory setting and the implication for training and agility actions (i.e., unplanned COD in response to a stimulus), which occur in open-skill conditions, such as team sports [18]. However, this study implemented an experimental approach to the investigation of the turning phase in several team sports players. The present investigation suggests that the penultimate, final, and first accelerating foot contacts should be assessed for a comprehensive understanding of the turning phase. Together with previous research [15,16,17,18,19,21], we have evidence from four foot contacts characterizing the 180° COD performance. However, further research can be encouraged to extend the analysis of other foot contacts and their contribution to completion time.

## 5. Conclusions

The present study provided a characterization of the turning phase during the modified 505 test, demonstrating that each of the three foot contacts can play an important role in COD performance. In particular, the PFC and FFC are considered critical foot contacts for the deceleration of the body and the preparation for reacceleration in the new direction. Conversely, the AFC is the first foot contact after turning when the horizontal velocity of the center of mass is zero and the reacceleration in the new direction has to be executed. Among several spatial–temporal and kinetic variables, the propulsive horizontal GRF of the AFC can be emphasized, as it is able to indicate faster performers and the high-playing-level group and predict faster completion times in both braking/propulsive and only-propulsive trials. The findings of this study can be translated to practical implications for training. An important component of COD speed is the deceleration phase, meaning that athletes should have a high braking ability. Furthermore, an efficient COD performance can be achieved with a fast transition from the deceleration phase to the acceleration phase, meaning a fast coupling of eccentric and concentric muscle action, which is an expression of the SSC. Therefore, to achieve these goals, it is confirmed that training programs should be implemented with strength training [39]. Due to the contribution of eccentric, concentric, dynamic, and isometric strength to COD performance [38], a variety of exercises may be proposed in order to enhance the ability to change momentum and coordinate body movement within the constraints of the activity [39]. Among the several forms of strength training, the application of eccentric training for the improvement of the braking ability of athletes has been recently emphasized. Eccentric exercises can be executed under several conditions and modalities, as summarized in recent reviews, and are strongly recommended for the wide spectrum of training adaptations [40,41,42]. Moreover, exercises for the application of the SSC should be implemented. Change-of-direction speed can surely benefit from a variety of exercises considering machine-based, elastic band, and plyometric exercises, particularly when executed in a unilateral, multiplanar, and multidirectional fashion, to replicate the demands of team sports performance. Therefore, to be faster in performing directional changes, athletes should follow these recommendations.

## Figures and Tables

**Table 1:** Comparisons of spatial–temporal and kinetic variables during the turning phase between braking/propulsive and only-propulsive trials (mean ± SD).

	Variables	Braking/Propulsive Trials	Only-Propulsive Trials	*p* (ES)
Penultimate Foot Contact	Total CT (s)	0.385 ± 0.103	0.391 ± 0.13	0.677 (0.07)
	Braking VGRF (N/kg)	27.0 ± 7.3	28.0 ± 7.4	0.018 (0.41)
	Braking HGRF (N/kg)	15.1 ± 4.1	15.5 ± 4.1	0.137 (0.25)
	Braking VImp (N·s/kg)	2.3 ± 0.5	2.3 ± 0.4	0.654 (0.08)
	Braking HImp (N·s/kg)	1.4 ± 0.3	1.4 ± 0.3	0.935 (0.01)
	Braking resultant GRF (N/kg)	30.5 ± 8	31.6 ± 8.1	0.012 (0.44)
	Step length (cm)	113.7 ± 33.9	116.7 ± 34.2	0.145 (0.24)
Final Foot Contact	Braking CT (s)	0.223 ± 0.036	0.230 ± 0.047	0.297 (0.18)
	Propulsive CT (s)	0.300 ± 0.055	0.301 ± 0.069	0.937 (0.01)
	Total CT (s)	0.524 ± 0.07	0.534 ± 0.083	0.303 (0.17)
	Braking VGRF (N/kg)	21.5 ± 4.9	21.9 ± 4.3	0.510 (0.11)
	Propulsive VGRF (N/kg)	14.3 ± 1.1	14.4 ± 1.3	0.260 (0.19)
	Braking HGRF (N/kg)	16.3 ± 3	16.7 ± 2.5	0.148 (0.24)
	Propulsive HGRF (N/kg)	11.1 ± 1.6	10.9 ± 1.4	0.177 (0.23)
	Braking VImp (N·s/kg)	2.7 ± 0.4	2.8 ± 0.5	0.023 (0.40)
	Propulsive VImp (N·s/kg)	3.0 ± 0.5	3.1 ± 0.6	0.490 (0.12)
	Total VImp (N·s/kg)	5.7 ± 0.6	5.9 ± 0.7	0.021 (0.40)
	Braking HImp (N·s/kg)	2.2 ± 0.4	2.3 ± 0.4	0.013 (0.41)
	Propulsive HImp (N·s/kg)	2.3 ± 0.3	2.2 ± 0.4	0.316 (0.17)
	Total HImp (N·s/kg)	4.4 ± 0.5	4.5 ± 0.5	0.184 (0.22)
	Braking resultant GRF (N/kg)	27.0 ± 5.4	27.3 ± 4.8	0.632 (0.08)
	Propulsive resultant GRF (N/kg)	18.1 ± 1.7	18.7 ± 4	0.312 (0.17)
	Step length (cm)	91.0 ± 13.5	92.3 ± 11.9	0.425 (0.13)
First Accelerating Foot Contact	Braking CT (s)	0.089 ± 0.032	N/A	N/A
	Propulsive CT (s)	0.224 ± 0.048	N/A	N/A
	Total CT (s)	0.313 ± 0.065	0.301 ± 0.06	0.219 (0.21)
	Braking HGRF (N/kg)	8.9 ± 2.8	N/A	N/A
	Propulsive VGRF (N/kg)	16.1 ± 1.9	16.7 ± 1.9	0.007 (0.48)
	Braking HGRF (N/kg)	4.6 ± 1.6	N/A	N/A
	Propulsive HGRF (N/kg)	9.0 ± 1.4	9.3 ± 1.3	0.016 (0.42)
	Braking VImp (N·s/kg)	0.5 ± 0.2	N/A	N/A
	Propulsive VImp (N·s/kg)	2.2 ± 0.4	N/A	N/A
	Total VImp (N·s/kg)	2.7 ± 0.3	2.6 ± 0.3	0.005 (0.50)
	Braking HImp (N·s/kg)	0.2 ± 0.1	N/A	N/A
	Propulsive HImp (N·s/kg)	1.2 ± 0.2	N/A	N/A
	Total HImp (N·s/kg)	1.4 ± 0.1	1.4 ± 0.2	0.162 (0.02)
	Braking resultant GRF (N/kg)	9.6 ± 3.2	N/A	N/A
	Propulsive resultant GRF (N/kg)	18.4 ± 2.2	19.0 ± 2	0.020 (0.41)
	Step length (cm)	81.8 ± 9.6	77.9 ± 10.8	0.024 (0.38)

Note: AU = arbitrary unit; CT = contact time; ES = effect size; HGRF = horizontal ground reaction force; HImp = horizontal impulse; N/A = not available; VGRF = vertical ground reaction force; VImp = vertical impulse.

**Table 2:** Comparison of kinetic variables during the turning phase between faster and slower performers for braking/propulsive and only-propulsive trials (mean ± SD).

	Variables	Braking/Propulsive Trials			Only-Propulsive Trials		
		Faster	Slower	*p* (ES)	Faster	Slower	*p* (ES)
Penultimate Foot Contact	Total CT (s)	0.392 ± 0.068	0.355 ± 0.084	0.119 (0.48)	0.403 ± 0.084	0.352 ± 0.091	0.116 (0.58)
	Braking VGRF (N/kg)	29.2 ± 8.8	24.1 ± 7.1	0.046 (0.62)	29.4 ± 7.6	25.7 ± 6.3	0.163 (0.52)
	Braking HGRF (N/kg)	16.2 ± 4	12.4 ± 4.2	0.005 (0.91)	16.7 ± 3.6	12.9 ± 3.6	0.005 (1.06)
	Braking VImp (N·s/kg)	2.3 ± 0.3	2.1 ± 0.5	0.066 (0.57)	2.2 ± 0.4	2.4 ± 0.5	0.201 (−0.47)
	Braking HImp (N·s/kg)	1.5 ± 0.2	1.2 ± 0.3	<0.001 (1.3)	1.4 ± 0.2	1.3 ± 0.2	0.145 (0.53)
	Braking resultant GRF (N/kg)	32.4 ± 8.9	26.7 ± 7.7	0.03 (0.68)	33.1 ± 8	28.5 ± 6.8	0.099 (0.62)
Final Foot Contact	Braking CT (s)	0.227 ± 0.04	0.223 ± 0.036	0.724 (0.11)	0.231 ± 0.056	0.233 ± 0.047	0.915 (−0.04)
	Propulsive CT (s)	0.277 ± 0.044	0.330 ± 0.055	0.001 (−1.1)	0.275 ± 0.049	0.317 ± 0.069	0.055 (−0.72)
	Total CT (s)	0.504 ± 0.059	0.552 ± 0.074	0.02 (−0.73)	0.506 ± 0.069	0.556 ± 0.094	0.091 (−0.63)
	Braking VGRF (N/kg)	20.8 ± 3.9	20.5 ± 3.3	0.789 (0.08)	21.3 ± 3.5	21.4 ± 5.2	0.927 (−0.03)
	Propulsive VGRF (N/kg)	14.4 ± 1	13.9 ± 0.9	0.088 (0.53)	14.7 ± 1.2	15.2 ± 0.9	0.204 (−0.47)
	Braking HGRF (N/kg)	17.1 ± 2.7	15.0 ± 2.5	0.012 (0.82)	17.4 ± 2.5	14.5 ± 1.9	0.001 (1.29)
	Propulsive HGRF (N/kg)	11.9 ± 1.6	10.0 ± 0.9	<0.001 (1.45)	11.7 ± 1.5	10.6 ± 0.9	0.031 (0.79)
	Braking VImp (N·s/kg)	2.7 ± 0.4	2.6 ± 0.4	0.316 (0.31)	2.9 ± 0.6	2.7 ± 0.6	0.567 (0.21)
	Propulsive VImp (N·s/kg)	2.8 ± 0.4	3.3 ± 0.5	<0.001 (−1.17)	2.9 ± 0.4	3.4 ± 0.7	0.025 (−0.86)
	Total VImp (N·s/kg)	5.5 ± 0.5	5.9 ± 0.7	0.043 (−0.63)	5.8 ± 0.5	6.1 ± 0.9	0.19 (−0.48)
	Braking HImp (N·s/kg)	2.3 ± 0.4	1.9 ± 0.4	0.001 (1.08)	2.4 ± 0.4	1.9 ± 0.4	0.004 (1.11)
	Propulsive HImp (N·s/kg)	2.2 ± 0.3	2.3 ± 0.3	0.231 (−0.38)	2.3 ± 0.3	2.3 ± 0.4	0.918 (−0.04)
	Total HImp (N·s/kg)	4.5 ± 0.4	4.3 ± 0.5	0.054 (0.61)	4.7 ± 0.3	4.2 ± 0.6	0.006 (1.03)
	Braking resultant GRF (N/kg)	26.7 ± 4.6	25.2 ± 3.8	0.248 (0.35)	27.4 ± 4.3	25.7 ± 5.1	0.333 (0.36)
	Propulsive resultant GRF (N/kg)	18.6 ± 1.7	17.1 ± 1.2	0.002 (1)	18.7 ± 1.8	18.6 ± 1.1	0.869 (0.06)
First Accelerating Foot contact	Braking CT (s)	0.089 ± 0.035	0.088 ± 0.027	0.913 (0.03)	N/A	N/A	N/A
	Propulsive CT (s)	0.203 ± 0.03	0.230 ± 0.038	0.012 (−0.8)	N/A	N/A	N/A
	Total CT (s)	0.293 ± 0.052	0.316 ± 0.047	0.133 (−0.46)	0.299 ± 0.058	0.270 ± 0.048	0.143 (0.54)
	Braking VGRF (N/kg)	8.8 ± 2.9	9.3 ± 2.9	0.579 (−0.17)	N/A	N/A	N/A
	Propulsive VGRF (N/kg)	16.0 ± 2	15.2 ± 1.6	0.162 (0.43)	17.0 ± 2.1	17.0 ± 1.5	0.996 (0)
	Braking HGRF (N/kg)	4.8 ± 1.5	4.4 ± 1.8	0.418 (0.25)	N/A	N/A	N/A
	Propulsive HGRF (N/kg)	9.6 ± 1.1	7.6 ± 1	<0.001 (1.82)	10.0 ± 1	8.7 ± 1.2	0.003 (1.12)
	Braking VImp (N·s/kg)	0.4 ± 0.2	0.5 ± 0.2	0.133 (−0.46)	N/A	N/A	N/A
	Propulsive VImp (N·s/kg)	2.1 ± 0.3	2.3 ± 0.4	0.022 (−0.72)	2.5 ± 0.3	2.5 ± 0.2	0.705 (−0.14)
	Total VImp (N·s/kg)	2.5 ± 0.3	2.8 ± 0.4	0.003 (−0.96)	N/A	N/A	N/A
	Braking HImp (N·s/kg)	0.2 ± 0.1	0.2 ± 0.1	0.53 (−0.2)	N/A	N/A	N/A
	Propulsive HImp (N·s/kg)	1.2 ± 0.1	1.1 ± 0.2	0.014 (0.79)	1.4 ± 0.2	1.3 ± 0.1	0.004 (1.09)
	Total HImp (N·s/kg)	1.5 ± 0.1	1.3 ± 0.3	0.045 (0.64)	N/A	N/A	N/A
	Braking resultant GRF (N/kg)	9.9 ± 3	9.5 ± 3.7	0.637 (0.14)	N/A	N/A	N/A
	Propulsive resultant GRF (N/kg)	18.6 ± 2.4	16.9 ± 1.7	0.013 (0.78)	19.6 ± 2.2	18.9 ± 1.7	0.362 (0.33)

Note: AU = arbitrary unit; CT = contact time; ES = effect size; HGRF = horizontal ground reaction force; HImp = horizontal impulse; N/A = not available; VGRF = vertical ground reaction force; VImp = vertical impulse.

**Table 3:** Comparison of kinetic variables during the turning phase between the high- and low-playing-level groups for braking/propulsive and only-propulsive trials (mean ± SD).

	Variables	Braking/Propulsive Trials			Only-Propulsive Trials		
		High Playing Level	Low Playing Level	*p* (ES)	High Playing Level	Low Playing Level	*p* (ES)
Penultimate Foot Contact	Total CT (s)	0.381 ± 0.081	0.383 ± 0.1	0.902 (−0.03)	0.378 ± 0.098	0.405 ± 0.141	0.454 (−0.22)
	Braking VGRF (N/kg)	31.2 ± 7.3	23.5 ± 7.1	<0.001 (1.08)	31.4 ± 5.5	24.2 ± 7.1	<0.001 (1.14)
	Braking HGRF (N/kg)	16.6 ± 3.3	13.2 ± 4.3	0.001 (0.86)	16.9 ± 3	13.5 ± 4.4	0.002 (0.91)
	Braking VImp (N·s/kg)	2.5 ± 0.3	2.2 ± 0.5	0.007 (0.68)	2.4 ± 0.4	2.2 ± 0.5	0.144 (0.43)
	Braking HImp (N·s/kg)	1.5 ± 0.2	1.3 ± 0.3	0.001 (0.85)	1.4 ± 0.2	1.3 ± 0.3	0.076 (0.51)
	Braking resultant GRF (N/kg)	34.4 ± 7.4	26.4 ± 7.8	<0.001 (1.05)	34.9 ± 5.9	27.6 ± 8.1	0.001 (1.05)
Final Foot Contact	Braking CT (s)	0.233 ± 0.031	0.216 ± 0.043	0.081 (0.43)	0.239 ± 0.05	0.215 ± 0.035	0.062 (0.56)
	Propulsive CT (s)	0.298 ± 0.037	0.313 ± 0.059	0.225 (−0.30)	0.295 ± 0.058	0.308 ± 0.076	0.531 (−0.18)
	Total CT (s)	0.531 ± 0.047	0.528 ± 0.071	0.858 (0.04)	0.535 ± 0.072	0.527 ± 0.093	0.745 (0.10)
	Braking VGRF (N/kg)	22.3 ± 5.2	20.2 ± 3	0.037 (0.52)	23.4 ± 5.3	20.3 ± 2.7	0.017 (0.72)
	Propulsive VGRF (N/kg)	14.7 ± 1	13.8 ± 1.2	0.001 (0.81)	14.9 ± 1.2	14.3 ± 1.3	0.088 (0.51)
	Braking HGRF (N/kg)	16.5 ± 2.5	15.8 ± 2.7	0.246 (0.29)	17.0 ± 2.5	16.0 ± 2.5	0.190 (0.38)
	Propulsive HGRF (N/kg)	11.5 ± 1.3	10.4 ± 1.4	0.001 (0.87)	11.2 ± 1.3	10.7 ± 1.3	0.151 (0.41)
	Braking VImp (N·s/kg)	2.8 ± 0.3	2.6 ± 0.5	0.013 (0.62)	3.0 ± 0.5	2.6 ± 0.4	0.008 (0.81)
	Propulsive VImp (N·s/kg)	3 ± 0.4	3.1 ± 0.6	0.386 (−0.213)	3.1 ± 0.6	3.2 ± 0.7	0.546 (−0.18)
	Total VImp (N·s/kg)	5.8 ± 0.5	5.7 ± 0.6	0.258 (0.28)	6.0 ± 0.6	5.8 ± 0.8	0.212 (0.37)
	Braking HImp (N·s/kg)	2.3 ± 0.3	2.0 ± 0.4	0.001 (0.83)	2.4 ± 0.4	2.0 ± 0.4	0.004 (0.86)
	Propulsive HImp (N·s/kg)	2.3 ± 0.3	2.2 ± 0.3	0.579 (0.14)	2.3 ± 0.4	2.2 ± 0.4	0.661 (0.12)
	Total HImp (N·s/kg)	4.6 ± 0.4	4.3 ± 0.5	0.002 (0.81)	4.7 ± 0.4	4.3 ± 0.5	0.002 (0.93)
	Braking resultant GRF (N/kg)	27.7 ± 5.4	25.5 ± 3.7	0.05 (0.49)	28.8 ± 5.5	25.5 ± 3.3	0.016 (0.73)
	Propulsive resultant GRF (N/kg)	18.7 ± 1.3	17.1 ± 1.7	<0.001 (1.01)	19.6 ± 4.5	17.8 ± 1.7	0.076 (0.53)
First Accelerating Foot Contact	Braking CT (s)	0.090 ± 0.037	0.087 ± 0.029	0.745 (0.08)	N/A	N/A	N/A
	Propulsive CT (s)	0.202 ± 0.03	0.237 ± 0.053	0.003 (−0.77)	N/A	N/A	N/A
	Total CT (s)	0.290 ± 0.055	0.324 ± 0.06	0.021 (−0.58)	0.297 ± 0.066	0.302 ± 0.053	0.780 (−0.08)
	Braking VGRF (N/kg)	9.6 ± 2.4	8.4 ± 3	0.07 (0.45)	N/A	N/A	N/A
	Propulsive VGRF (N/kg)	16.3 ± 2	15.3 ± 1.6	0.025 (0.56)	16.6 ± 2.1	16.6 ± 1.4	0.952 (−0.02)
	Braking HGRF (N/kg)	5.2 ± 1.3	4.2 ± 1.6	0.009 (0.67)	N/A	N/A	N/A
	Propulsive HGRF (N/kg)	9.6 ± 1.2	8.1 ± 1.3	<0.001 (1.19)	9.6 ± 1.2	8.7 ± 1.1	0.008 (0.78)
	Braking VImp (N·s/kg)	0.5 ± 0.2	0.4 ± 0.2	0.537 (0.15)	N/A	N/A	N/A
	Propulsive VImp (N·s/kg)	2.2 ± 0.3	2.3 ± 0.4	0.056 (−0.47)	2.5 ± 0.3	2.6 ± 0.3	0.513 (−0.19)
	Total VImp (N·s/kg)	2.6 ± 0.3	2.8 ± 0.4	0.07 (0.45)	N/A	N/A	N/A
	Braking HImp (N·s/kg)	0.2 ± 0.1	0.2 ± 0.1	0.282 (0.27)	N/A	N/A	N/A
	Propulsive HImp (N·s/kg)	1.2 ± 0.1	1.2 ± 0.2	0.099 (0.42)	1.4 ± 0.2	1.3 ± 0.1	0.04 (0.6)
	Total HImp (N·s/kg)	1.5 ± 0.1	1.4 ± 0.2	0.015 (0.62)	N/A	N/A	N/A
	Braking resultant GRF (N/kg)	10.8 ± 2.5	8.8 ± 3.3	0.007 (0.67)	N/A	N/A	N/A
	Propulsive resultant GRF (N/kg)	18.8 ± 2.2	17.2 ± 1.9	0.001 (0.83)	19 ± 2.2	18.8 ± 1.6	0.623 (0.14)

Note: AU = arbitrary unit; CT = contact time; ES = effect size; HGRF = horizontal ground reaction force; HImp = horizontal impulse; N/A = not available; VGRF = vertical ground reaction force; VImp = vertical impulse.

**Table 4:** Models derived from the stepwise multiple regression analysis.

	Model	R	R^2^	Adjusted R^2^	SEE	F	*p*
Braking/Propulsive Trials	1	0.708	0.501	0.493	0.100	65.3	<0.001
	2	0.763	0.582	0.569	0.092	44.5	<0.001
	3	0.843	0.711	0.697	0.077	51.6	<0.001
	4	0.856	0.733	0.716	0.075	42.6	<0.001
	5	0.866	0.750	0.730	0.073	36.7	<0.001
Only-Propulsive Trials	1	0.536	0.287	0.271	0.123	17.3	<0.001
	2	0.726	0.527	0.504	0.101	23.4	<0.001
	3	0.813	0.661	0.636	0.087	26.6	<0.001
	4	0.834	0.695	0.665	0.083	22.8	<0.001
	5	0.857	0.734	0.700	0.079	21.5	<0.001
	6	0.873	0.762	0.725	0.075	20.3	<0.001

Note: For braking/propulsive trials: 1. FFC propulsive HGRF; 2. FFC propulsive HGRF, AFC propulsive HGRF; 3. FFC propulsive HGRF, AFC propulsive HGRF, FFC propulsive VGRF; 4. FFC propulsive HGRF, AFC propulsive HGRF, FFC propulsive VGRF, AFC Total VImp; 5. FFC propulsive HGRF, AFC propulsive HGRF, FFC propulsive VGRF, AFC Total VImp, AFC step length. For only-propulsive trials: 1. Approach velocity; 2. Approach velocity, FFC braking HGRF; 3. Approach velocity, FFC braking HGRF, FFC braking VGRF; 4. Approach velocity, FFC braking HGRF, FFC braking VGRF, AFC propulsive HGRF; 5. Approach velocity, FFC braking HGRF, FFC braking VGRF, AFC propulsive HGRF, FFC total CT; 6. Approach velocity, FFC braking HGRF, FFC braking VGRF, AFC propulsive HGRF, FFC total CT, AFC propulsive VGRF. AFC = first accelerating foot contact; CT = contact time; FFC = final foot contact; HGRF = horizontal ground reaction force; SEE = standard error of estimate; VGRF = vertical ground reaction force; VImp = vertical impulse.

**Table 5:** Predictive variables of completion time.

		Unstandardized Coefficients		Standardized Coefficients		95% CI for B		Correlations		Collinearity Statistics	
	Model	B	Std. Error	Beta	t (*p*)	Lower Bound	Upper Bound	Partial *	Part ^#^	Tolerance	VIF
Braking/Propulsive Trials	(Constant)	3.087	0.174		17.721 (<0.001)	2.738	3.435				
	FFC propulsive HGRF	−0.076	0.009	−0.771	−8.520 (<0.001)	−0.093	−0.058	−0.737	−0.545	0.500	2.001
	AFC propulsive HGRF	−0.049	0.008	−0.500	−6.147 (<0.001)	−0.065	−0.033	−0.618	−0.393	0.619	1.616
	FFC propulsive VGRF	0.061	0.011	0.522	5.409 (<0.001)	0.038	0.083	0.569	0.346	0.440	2.274
	AFC total VImp	0.081	0.029	0.199	2.771 (0.007)	0.023	0.139	0.334	0.177	0.796	1.257
	AFC step length	−0.002	0.001	−0.144	−2.051 (0.045)	−0.004	<−0.0001	−0.254	−0.131	0.831	1.203
Only-Propulsive Trials	(Constant)	3.522	0.208		16.904 (<0.001)	3.100	3.944				
	Approach velocity	−0.095	0.026	−0.321	−3.684 (0.001)	−0.147	−0.043	−0.051	−0.291	0.821	1.217
	FFC braking HGRF	−0.030	0.007	−0.551	−4.569 (<0.001)	−0.044	−0.017	−0.595	−0.361	0420	2.326
	FFC braking VGRF	0.009	0.004	0.274	2.180 (0.035)	0.001	0.016	0.333	0.172	0.397	2.516
	AFC propulsive HGRF	−0.048	0.014	−0.403	−3.543 (0.001)	−0.075	−0.021	−0.498	−0.280	0.484	2.064
	FFC total CT	0.402	0.178	0.217	2.253 (0.03)	0.041	0.762	0.343	0.178	0.675	1.482
	AFC propulsive VGRF	0.018	0.008	0.226	2.122 (0.04)	0.001	0.035	0.325	0.168	0.553	1.807

Note: AFC = first accelerating foot contact; CI = confidence interval; CT = contact time; FFC = final foot contact; HGRF = horizontal ground reaction force; VGRF = vertical ground reaction force; VImp = vertical impulse. * Shared contributions of the predictors. ^#^ Unique contributions of the predictors.

## Data Availability

The data presented in this study are available on request from the corresponding author. The data are not publicly available due to privacy or ethical restrictions.

## Supplementary Materials

The following are available online at https://www.mdpi.com/article/10.3390/ijerph18115519/s1. Graphical representation of the laboratory setting is available as supplementary material with Figure S1. Intraclass correlation coefficients with the 95% confidence intervals of the variables investigated are reported in Table S1. The analysis of the effect of leg preference on variables investigated is provided in Table S2.
