# Supplementary material for: The Biomechanical Characterization of the Turning Phase during a 180° Change of Direction
Source: Int J Environ Res Public Health. 2021 May 21;18(11):5519. doi: 10.3390/ijerph18115519 (PMC8196559; doi:10.3390/ijerph18115519)
Supplement: Supplementary file 1 [file ijerph-18-05519-s001.zip › Supplementary Material-TableS1.pdf]

Table S1. Intraclass correlation coefficients (ICC) with the 95% confidence intervals (CI) for the investigated variables.

	PFC	FFC	AFC
	ICC (95% CI)	ICC (95% CI)	ICC (95% CI)
Contact time	0.882 (0.833-0.920)	0.814 (0.732-0.876)	0.854 (0.810-0.890)
Braking vertical GRF	0.967 (0.953-0.978)	0.931 (0.901-0.953)	0.936 (0.894-0.963)
Propulsive vertical GRF	N/A	0.976 (0.966-0.983)	0.956 (0.938-0.970)
Braking horizontal GRF	0.975 (0.964-0.983)	0.962 (0.946-0.974)	0.962 (0.936-0.978)
Propulsive horizontal GRF	N/A	0.939 (0.913-0.958)	0.969 (0.956-0.979)
Total vertical impulse	0.976 (0.966-0.983)	0.954 (0.935-0.969)	0.935 (0.906-0.957)
Total horizontal impulse	0.975 (0.965-0.983)	0.978 (0.969-0.985)	0.947 (0.924-0.965)
Step length	0.942 (0.913-0.962)	0.845 (0.770-0.899)	0.816 (0.726-0.880)
Sprint time		0.910 (0.869-0.941)	
Approach velocity		0.909 (0.862-0.944)	

Note: AFC = first accelerating foot contact; FFC = final foot contact; GRF = ground reaction force; N/A = not available; PFC = penultimate foot contact.
