# Supplementary material for: The Biomechanical Characterization of the Turning Phase during a 180° Change of Direction
Source: Int J Environ Res Public Health. 2021 May 21;18(11):5519. doi: 10.3390/ijerph18115519 (PMC8196559; doi:10.3390/ijerph18115519)
Supplement: Supplementary file 1 [file ijerph-18-05519-s001.zip › Supplementary Material-TableS2.pdf]

Table S2. Comparisons of spatial/temporal and kinetic variables during the turning phase between stance and kicking leg (mean±SD).

	Braking/propulsive trials			Only-propulsive trials		
**Variables**	**Stance leg**	**Kicking leg**	**p (ES)**	**Stance leg**	**Kicking leg**	**p (ES)**
**Penultimate foot contact**						
Total CT (s)	0.392±0.087	0.379±0.092	0.092 (0.15)	0.400±0.123	0.382±0.131	0.229 (0.14)
Braking VGRF (N/kg)	27.1±7.9	25.6±7.5	0.182 (0.19)	29.2±6.7	27.3±7.5	0.228 (0.27)
Braking HGRF (N/kg)	14.9±4.5	14.5±3.9	0.452 (0.09)	16.5±4.7	15.0±4.0	0.257 (0.34)
Braking VImp (N·s/kg)	2.3±0.4	2.3±0.4	0.347 (0.13)	2.5±0.5	2.3±0.4	0.142 (0.44)
Braking HImp (N·s/kg)	1.4±0.2	1.4±0.2	0.187 (0.12)	1.4±0.2	1.3±0.2	0.086 (0.50)
Braking resultant GRF (N/kg)	30.7±8.5	29.4±8.5	0.303 (0.15)	33.0±7.6	30.9±8.3	0.226 (0.26)
Step length (cm)	121.6±34.9	114.9±32.9	0.085 (0.20)	115.3±33.9	109.9±31.9	0.185 (0.16)
**Final foot contact**						
Braking CT (s)	0.233±0.034	0.220±0.041	0.093 (0.35)	0.237±0.055	0.221±0.032	0.141 (0.36)
Propulsive CT (s)	0.308±0.047	0.308±0.052	0.964 (0.01)	0.302±0.076	0.300±0.068	0.920 (0.03)
Total CT (s)	0.541±0.056	0.527±0.055	0.088 (0.25)	0.539±0.086	0.527±0.078	0.421 (0.15)
Braking VGRF (N/kg)	20.7±3.4	20.9±3.6	0.709 (-0.06)	21.6±5.3	22.2±4.5	0.481 (-0.12)
Propulsive VGRF (N/kg)	14.0±1.1	14.2±1.3	0.229 (-0.17)	14.8±1.2	15.0±1.0	0.490 (-0.18)
Braking HGRF (N/kg)	16.2±2.4	16.2±2.5	0.976 (0.01)	16.8±2.5	17.0±2.5	0.701 (-0.08)
Propulsive HGRF (N/kg)	10.7±1.4	11.1±1.5	0.130 (-0.28)	10.7±1.2	11.2±1.4	0.064 (-0.38)
Braking VImp (N·s/kg)	2.8±0.4	2.6±0.5	0.196 (0.44)	2.8±0.6	2.8±0.4	0.791 (0.07)
Propulsive VImp (N·s/kg)	3.0±5.4	3.1±0.5	0.317 (-0.03)	3.2±0.7	3.2±0.6	0.980 (0.01)
Total VImp (N·s/kg)	5.8±0.5	5.7±0.6	0.774 (0.18)	6.0±0.6	6.0±0.6	0.786 (0.06)
Braking HImp (N·s/kg)	2.2±0.3	2.2±0.4	0.344 (0.16)	2.4±0.5	2.4±0.4	0.630 (0.14)
Propulsive HImp (N·s/kg)	2.2±0.3	2.3±0.3	0.181 (-0.33)	2.2±0.4	2.3±0.4	0.268 (-0.25)
Total HImp (N·s/kg)	4.5±0.4	4.5±0.5	0.559 (-0.06)	4.6±0.5	4.7±0.3	0.401 (-0.24)
Braking resultant GRF (N/kg)	26.0±3.9	26.2±4.1	0.790 (-0.05)	26.9±5.5	27.4±4.8	0.545 (-0.10)
Propulsive resultant GRF (N/kg)	17.5±1.5	17.9±1.8	0.194 (-0.24)	19.7±5.3	18.8±1.5	0.505 (0.23)
Step length (cm)	92.3±11.1	91.3±11.6	0.642 (0.09)	90.7±11.3	91.6±13.0	0.718 (-0.07)
**First accelerating foot contact**						
Braking CT (s)	0.085±0.023	0.092±0.037	0.061 (-0.23)	N/A	N/A	N/A
Propulsive CT (s)	0.226±0.055	0.220±0.043	0.394 (0.12)	N/A	N/A	N/A
Total CT (s)	0.307±0.061	0.313±0.062	0.572 (-0.10)	0.294±0.060	0.302±0.060	0.346 (-0.13)
Braking VGRF (N/kg)	9.1±3.1	8.7±2.6	0.227 (0.14)	N/A	N/A	N/A
Propulsive VGRF (N/kg)	15.8±1.9	15.8±1.9	0.951 (-0.01)	17.0±2.0	16.7±1.8	0.423 (0.16)
Braking HGRF (N/kg)	4.8±1.5	4.5±1.5	0.072 (0.20)	N/A	N/A	N/A
Propulsive HGRF (N/kg)	8.7±1.5	8.7±1.4	0.804 (-0.03)	9.3±1.4	9.1±1.2	0.297 (0.15)
Braking VImp (N·s/kg)	0.5±0.2	0.5±0.2	0.746 (-0.06)	N/A	N/A	N/A
Propulsive VImp (N·s/kg)	2.3±0.4	2.2±0.4	0.377 (0.25)	N/A	N/A	N/A
Total VImp (N·s/kg)	2.8±0.4	2.7±0.3	0.364 (0.28)	2.6±0.3	2.6±0.3	0.743 (0.09)
Braking HImp (N·s/kg)	0.2±0.1	0.2±0.1	0.695 (0.07)	N/A	N/A	N/A
Propulsive HImp (N·s/kg)	1.2±0.2	1.2±0.2	0.489 (0.13)	N/A	N/A	N/A
Total HImp (N·s/kg)	1.4±0.2	1.4±0.2	0.393 (0.1.4)	1.4±0.1	1.4±0.2	0.878 (0.04)
Braking resultant GRF (N/kg)	10.1±3.2	9.6±2.8	0.172 (0.17)	N/A	N/A	N/A
Propulsive resultant GRF (N/kg)	18.0±2.3	18.0±2.2	0.916 (0.15)	19.3±2.2	18.9±2.0	0.402 (0.19)
Step length (cm)	82.0±9.6	83.8±10.6	0.329 (-0.18)	76.3±7.1	79.6±9.2	0.090 (-0.40)

Note: AFC = first accelerating foot contact; CT = contact time; FFC = final foot contact; HGRF = horizontal ground reaction force; HImp = horizontal impulse; N/A = not available; PFC = penultimate foot contact; VGRF = vertical ground reaction force; VImp = vertical impulse. Completion time (stance leg: 2.76±0.14 s; kicking leg: 2.77±0.14 s; p = 399; ES = -0.07) and approach velocity (stance leg: 5.36±0.53 m/s; kicking leg: 5.43±0.40 m/s; p = 0.253; ES = -0.15).
