# Supplementary figures and images for: The Biomechanical Characterization of the Turning Phase during a 180° Change of Direction
Source: Int J Environ Res Public Health. 2021 May 21;18(11):5519. doi: 10.3390/ijerph18115519 (PMC8196559; doi:10.3390/ijerph18115519)

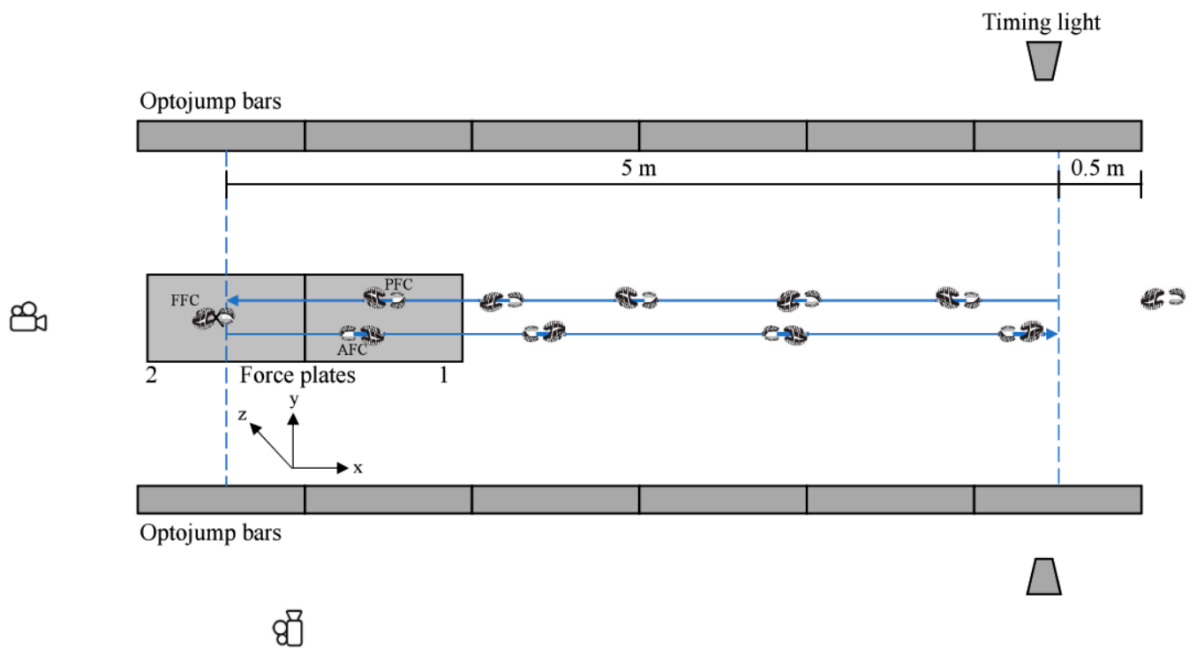

**Figure S1.** Graphical representation of the laboratory setting.

Supplement: Supplementary file 1 [file ijerph-18-05519-s001.zip › ijerph-1179348-Supplementary Figure.pdf]
